# Genetically Modified *Lactococcus lactis* Hypersecreting IL‐1Ra Improves Glucose Metabolism and Modulates the Gut Microbiota in an Obese Mouse Model

**DOI:** 10.1155/jdr/6006491

**Published:** 2026-02-01

**Authors:** Masahiro Yoda, Natsumi Nomura, Shoko Yoda, Mao Kagotani, Aito Murakami, Fu Namai, Tadashi Fujii, Takumi Tochio, Takashi Sato, Takeshi Shimosato

**Affiliations:** ^1^ Graduate School of Medicine, Science and Technology, Shinshu University, Nagano, Japan, shinshu-u.ac.jp; ^2^ Department of Agriculture, Graduate School of Science and Technology, Shinshu University, Nagano, Japan, shinshu-u.ac.jp; ^3^ Institute for Aqua Regeneration, Shinshu University, Nagano, Japan, shinshu-u.ac.jp; ^4^ Food and Feed Immunology Group, Laboratory of Animal Food Function, Graduate School of Agricultural Science, Tohoku University, Sendai, Miyagi, Japan, tohoku.ac.jp; ^5^ Institute for Biomedical Sciences, Shinshu University, Nagano, Japan, shinshu-u.ac.jp; ^6^ Department of Medical Research on Prebiotics and Probiotics, Fujita Health University, Toyoake, Aichi, Japan, fujita-hu.ac.jp; ^7^ BIOSIS Lab. Co., Ltd., Toyoake, Aichi, Japan; ^8^ HAKKOU JAPAN, Co., Ltd., Matsumoto, Nagano, Japan

**Keywords:** diet-induced obese mouse model, glucose metabolism, gmLAB, IL-1Ra, IL-1*β*, microbiota

## Abstract

Type 2 diabetes mellitus (T2DM) is a chronic metabolic disorder and typically develops later in life due to systemic dysfunction in metabolic homeostasis and various factors related to *β*‐cell inflammation. Interestingly, recent studies have proposed that intra‐islet expression of inflammatory cytokines, particularly interleukin (IL)‐1*β*, contributes to the pathogenesis of T2DM and have shown that blockade of IL‐1*β* signaling improves glycemia and *β*‐cell secretory function. We recently successfully constructed a genetically modified lactic acid bacteria (gmLAB) strain that hypersecretes recombinant mouse IL‐1 receptor antagonist (rmIL‐1Ra), that is, NZ‐IL1Ra. In this study, we investigated how NZ‐IL1Ra affects glucose metabolism using a mouse pancreatic *β*‐cell line and diet‐induced obese mouse model. We found that rmIL‐1Ra purified from NZ‐IL1Ra suppresses the expression of mouse pancreatic *β*‐cell genes related to inflammation. In addition, the results of oral glucose tolerance tests revealed that administration of NZ‐IL1Ra improves glucose metabolism, but the extent depends on the route of administration. Finally, microbiota analyses revealed increases in the abundances of two genera of Lachnospiraceae. These microbiota changes might also affect glucose metabolism in mice. Taken together, our results suggest that administration of NZ‐IL1Ra may be a useful tool for improving glucose metabolism.

## 1. Introduction

Diabetes mellitus, a chronic metabolic disorder characterized by abnormally high blood glucose levels, is a major public health problem. Two primary types of diabetes have been described: Type 1 diabetes mellitus and Type 2 diabetes mellitus (T2DM). Type 1 diabetes mellitus develops in the early stages of life due primarily to an autoimmune disorder. By contrast, T2DM typically develops later in life due to systemic dysfunction in metabolic homeostasis. Unlike Type 1 diabetes mellitus, T2DM is comparatively complex and involves numerous pathologic mechanisms. Over the past decade, several reports have suggested that islet inflammation may play a role in the functional defects observed in pancreatic *β*‐cells associated with the development of T2DM [1]. Research has suggested that intra‐islet expression of inflammatory cytokines, particularly interleukin (IL)‐1*β* [2], contributes to the pathogenesis of T2DM [3, 4].

The pro‐inflammatory activities of IL‐1*β* are mediated via binding to the interleukin‐1 receptor (IL‐1R) Type I [5]. However, these activities of IL‐1*β* are inhibited by several factors, such as interleukin‐1 receptor antagonist (IL‐1Ra) [6], which competitively inhibits the binding of IL‐1 to its receptor, IL‐1R [7–10]. A clinical trial showed that daily injection of a recombinant IL‐1Ra product, anakinra [11], improved manifestations in patients with systemic inflammation [12] and those with IL‐1Ra deficiency [13]. Another study showed that blockade of IL‐1 signaling using anakinra improves glycemia and *β*‐cell secretory function and reduces markers of systemic inflammation [14]. These data suggest that IL‐1*β* is a potential target for preserving *β*‐cell function and that IL‐1*β* blockade may be a useful therapeutic approach for treating T2DM [14, 15].

Interestingly, in our previous study, we successfully constructed a microbial therapeutic based on genetically modified lactic acid bacteria (gmLAB) [16, 17] strain that hypersecretes recombinant mouse interleukin‐1Ra (rmIL‐1Ra) and demonstrated amelioration of the disease activity index score in mice with acute colitis [18]. In that study, the rmIL‐1Ra–secreting gmLAB was used to treat intestinal inflammation; however, we did not investigate the effectiveness of the gmLAB in the treatment of T2DM or management of glucose metabolism, which is correlated with systemic inflammation. Thus, the present study further evaluated the bioactivity of our rmIL‐1Ra by examining the expression of inflammation‐related genes in a mouse pancreatic *β*‐cell line. In subsequent in vivo experiments, the rmIL‐1Ra–secreting gmLAB was administered to C57BL/6 mice, which were then subjected to analysis of inflammation‐related gene expression in the pancreas and the oral glucose tolerance test (OGTT). We also investigated the effect of gmLAB administration on the intestinal microbiome. The results of the present study indicated that the rmIL‐1Ra–secreting gmLAB may be useful for improving glucose metabolism.

## 2. Materials and Methods

### 2.1. Bacteria, Growth Conditions, and gmNZ9000 Strain for Mouse IL‐1Ra Gene Expression

As the host strain, we used NZ9000 (MobiTec, Goettingen, Germany), a derivative of *Lactococcus lactis* subsp. *cremoris* MG1363. M17 broth (BD Difco, Becton, Dickinson and Company, Sparks, Maryland, United States) supplemented with 0.5% glucose (GM17) was used for bacterial culture, and constructed gmLAB were cultured in GM17 containing 10 *μ*g/mL chloramphenicol (GM17cm). Strain gmNZ9000, which expresses mouse IL‐1Ra (NZ‐IL1Ra), was prepared as described in our previous study [18]. NZ‐VC [19], a derivative strain of NZ9000 harboring pNZ8148#2:SEC, was used as a vector control strain. Expression of recombinant genes was induced according to a previously described method [18] using nisin (MoBiTec) at 1.25 ng/mL, which was previously determined as the optimal concentration, followed by incubation for an additional 3 h.

### 2.2. Detection and Purification of mIL‐1Ra

Samples were prepared according to a previous report [19]. gmLAB were cultured in 2 or 1000 mL of GM17cm for mIL‐1Ra detection or purification, respectively. Isolated proteins were analyzed using sodium dodecyl sulfate–polyacrylamide gel electrophoresis immunoblotting with mouse anti–His‐tag antibody (Ab) (BioLegend, San Diego, California, United States) and horseradish peroxidase–conjugated goat anti‐mouse IgG (Sigma‐Aldrich, St. Louis, Missouri, United States). The blots were reacted with ECL Prime Western Blotting Detection Reagent (Cytiva, Marlborough, Massachusetts, United States), and bands of interest were visualized using a Lumino image analyzer (ImageQuant LAS 500, Cytiva). rIL‐6R*α*scFv was purified using a HisTrap HP column (1 mL, Cytiva) and fast protein liquid chromatography system (AKTA pure 25, Cytiva) according to a previous report [18].

### 2.3. Culture Conditions for *β*‐TC‐tet Cells

*β*‐TC‐tet mouse pancreatic *β*‐cells (ATCC, Manassas, Virginia, United States) [20, 21] were cultured in cell culture dishes (BM Equipment Co., Ltd., Tokyo, Japan) in Dulbecco′s modified eagle medium (DMEM) (Thermo Fisher Scientific, Rockford, Illinois, United States) containing 10% fetal bovine serum (GE Healthcare), 100 U/mL penicillin, and 100 *μ*g/mL streptomycin (Nacalai Tesque) (complete DMEM) at 37°C in a humidified incubator supplied with 5% carbon dioxide. Cells were used for experiments upon reaching 80%–90% coverage of the dish growth surface.

### 2.4. Immunofluorescence Analysis of *β*‐Cells to Confirm IL‐1R Expression

For immunofluorescence microscopy, cells were cultured for 2 h on cover glasses in a 24‐well plate (Nippon Genetics, Tokyo, Japan) at a density of 2 × 10^6^ cells per well and then stimulated for 24 h with IL‐1*β* (1 *μ*g/mL) (R&D Systems, Minneapolis, Minnesota, United States). After the 24‐h stimulation, the cells were fixed for 10 min in 10% neutral buffered formalin solution (Fujifilm Wako Pure Chemical Corp., Osaka, Japan). Expression of IL‐1R was confirmed by incubating cells for 1 h with 1:100‐diluted PE anti–mouse IL‐1R/CD121a Ab (clone JAMA‐147, catalog #113505, BioLegend) or PE Armenian Hamster IgG Isotype Ctrl Ab (clone HTK888, catalog #400907, BioLegend). Fluorescence was observed using a BZ‐X800 fluorescence microscope (Keyence, Osaka, Japan).

### 2.5. Bioactivity Assay of Purified rmIL‐1Ra

*β*‐TC‐tet cells were seeded in a 24‐well plate (Nippon Genetics) at a density of 1 × 10^6^ cells per well and cultured for 2 h. The cells were then washed with phosphate‐buffered saline (PBS) and stimulated for 2 h with IL‐1*β* (0 or 5 ng/mL) (R&D Systems), IL‐1Ra (commercial) (50 ng/mL, R&D Systems), or rIL‐1Ra purified from NZ‐IL1Ra (50, 100 ng/mL).

### 2.6. RNA Extraction and Real‐Time Quantitative Reverse Transcription‐PCR (RT‐qPCR)

Total RNA was extracted from cells or tissue specimens using Nucleospin RNA (Macherey‐Nagel, North Rhine‐Westphalia, Germany). cDNA was synthesized using a PrimeScript RT reagent kit (Perfect Real Time) (TaKaRa, Shiga, Japan). RT‐qPCR analysis was performed using a Thermal Cycler Dice Real‐Time System (Takara Bio, Shiga, Japan) and TB Green Premix Ex Taq II (Takara Bio) with primers specific for mouse *Il6*, *Nfkb*, *Tnfa*, *Il1b*, and *Il17a* (Takara Bio). Data were normalized according to the expression of *β*‐actin (housekeeping gene).

### 2.7. Mice

Four‐week‐old C57BL/6 male mice were obtained from Japan SLC, Inc. (Hamamatsu, Japan) and housed under controlled humidity and temperature conditions with a 12‐h light–dark cycle and ad libitum access to water and pellet food MF (Oriental Yeast Co., Tokyo, Japan) during acclimatization. All animal procedures were performed according to the regulations for Animal Experimentation of Shinshu University (Approval No. 023095).

### 2.8. NZ‐IL1Ra Administration

After 1 week of acclimatization, mice were fed a high‐fat diet (HFD) (60 kcal% fat, D12492, research diet, New Brunswick, New Jersey, United States) as the diet‐induced obesity group (HFD group) or control diet (CD) (10 kcal% fat, D12450B, research diet) as the CD group. Mice in the HFD group were randomly assigned into five subgroups: PBS oral administration (p.o.) (*n* = 16) (PBS), NZ‐VC p.o. (*n* = 15), NZ‐IL1Ra p.o. (*n* = 14), NZ‐VC intraperitoneal (i.p.) (*n* = 3), and NZ‐IL1Ra i.p. (*n* = 3). Mice in the CD group were assigned into two subgroups: no treatment (*n* = 12) (CD‐NT) or NZ‐IL1Ra p.o. (*n* = 3) (CD‐NZ‐IL1Ra p.o.).

NZ‐VC and NZ‐IL1Ra cells were cultured, and gene expression was induced as described above. After nisin induction, bacterial cells and culture supernatants were separated by centrifugation at 8000 × *g* and 4°C for 5 min. The bacterial cells were resuspended in water, frozen for 24 h at −80°C, freeze‐dried (Yamato Scientific Co., Tokyo, Japan) for 24 h, and then resuspended in PBS at 5.0 × 10^9^ cells/200 *μ*L. Each mouse was administered 5.0 × 10^9^ NZ‐IL1Ra or NZ‐VC cells/200 *μ*L five times per week (total 35 times) for 7 weeks via gavage or i.p. injection. Body weight was measured weekly. A total of 7 weeks after the first dosing, OGTTs were performed.

### 2.9. OGTT

Glucose tolerance was evaluated by injecting mice p.o. with 2 g/kg of glucose (Nacalai Tesque) after a 16‐h fast at 7 weeks after the first administration. Blood glucose was measured via tail vein bleeding at defined time points (0, 15, 30, 60, 90, and 120 min after glucose administration) using an Accu‐CHEK Aviva monitor (Roche DC Japan, Tokyo, Japan). The area under the curve of blood glucose concentration from 0 min to 120 min (AUC_0–120 min_) was calculated using GraphPad Prism software (Version 7, GraphPad, San Diego, California, United States).

### 2.10. Extraction of RNA From Tissue Samples

Mice were sacrificed under anesthesia, and then the pancreas, spleen, and entire colon were collected. Tissue samples were homogenized using a Beads Crusher *μ*T‐12 operated at 2200 rpm for 150 s. RNA extraction, cDNA synthesis, and qPCR analyses were performed as described above.

### 2.11. Quantitation of rmIL‐1Ra in the Pancreas of Male C57BL/6 Mice

After 1 week of acclimatization, 18 mice (7‐week‐old male C57BL/6 mice) were randomly assigned to three groups: no treatment, p.o. administration of 5.0 × 10^9^ NZ‐IL1Ra cells/200 *μ*L PBS, and i.p. administration of 5.0 × 10^9^ NZ‐IL1Ra cells‐crushed/200 *μ*L PBS. Treatments were administered five times per day at 30‐min intervals. At 1.5 h after the final (fifth) treatment, all mice were sacrificed under anesthesia. The pancreas was collected and homogenized using a Beads Crusher *μ*T‐12 (lT‐12, TAITEC) operated at 3200 rpm for 1 min. After homogenization, the supernatant was separated by centrifugation at 8000 × *g* and 4°C for 5 min. The total protein concentration of the supernatant was determined using a BCA Protein Assay kit (Thermo Fisher Scientific, Rockford, Illinois, United States) according to the manufacturer′s instructions, and the concentration was subsequently adjusted to 2.0 mg/mL with PBS. The concentration of rmIL‐1Ra in the supernatant samples was determined using a mouse IL‐1ra/IL‐1F3 Quantikine ELISA kit (R&D Systems) according to the manufacturer′s instructions.

### 2.12. Gut Microbiota Analysis

Cecum contents were collected after the OGTTs, and DNA was extracted at the end of administration using a QIAamp Fast DNA Stool Mini kit (Qiagen, Valencia, California, United States) and Zirco Prep Mini tube (Nippon Genetics). The V3–V4 region of 16S rRNA was amplified using Tks Gflex DNA polymerase (Takara Bio). DNA was purified using AMPure XP Beads (Nippon Genetics), and Index PCR was performed using a NexteraXT Index kit (Illumina, San Diego, California, United States) in a 96‐well plate. Index PCR products were purified using AMPure XP Beads, as described above. The resulting DNA library was sequenced using a MiSeq system (Illumina). The Quantitative Insights into Microbial Ecology platform, Version 2 (Qiime2) [22], was used for microbiota analyses. The resulting amplicon data were filtered, denoised, and merged using the divisive amplicon sequence variant (ASV) table. Taxonomic annotation was performed using a classifier based on the Silva 138 database [23]. *α*‐Diversity was determined based on Faith′s phylogenetic diversity (PD), observed ASVs, and the Shannon diversity index. Principal coordinate analysis (PCoA) and significance difference testing using the *β*‐diversity (Bray–Curtis) index were also performed.

### 2.13. Statistical Analysis

Statistical analyses were performed using GraphPad Prism software (GraphPad). One‐way analysis of variance (ANOVA) and Tukey–Kramer tests were used to determine the significance of differences in RT‐qPCR data for bioactivity assays and quantitation of rmIL‐1Ra in the pancreas. One‐way ANOVA and Dunnett′s multiple comparisons test were used to determine the significance of differences in OGTT AUC values. Two‐way mixed models and Dunnett′s multiple comparisons test were used to determine the significance of differences between OGTT time‐course data. PCoA and significance difference testing using the *β*‐diversity index were also performed using adonis (permutational multivariate analysis of variance: PERMANOVA) in Qiime2. Differences were considered significant at *p* < 0.05.

## 3. Results

### 3.1. Confirmation of mIL‐1Ra Expression by gmLAB

**Figure 1:**
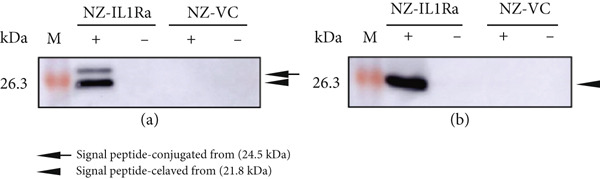
Detection of rmIL‐1Ra produced by NZ‐IL1Ra. (a) Western blotting of rmIL‐1Ra in the cell extract. (b) Western blotting of rmIL‐1Ra in the culture supernatant. An anti–His‐tag antibody was used for detection in both gmLABs (NZ‐IL1Ra and NZ‐VC). Arrow indicates the signal peptide–conjugated form of rmIL‐1Ra (24.5 kDa). Arrowheads indicate the signal peptide–cleaved form of rmIL‐1Ra (21.8 kDa). +, with nisin; −, without nisin; M, molecular mass marker (kDa).

In this study, we used the genetically modified strain NZ9000, an mIL‐1Ra–producing gmLAB (designated NZ‐IL1Ra), which was previously constructed by Namai et al. [18]. Before initiation of the study, expression of the recombinant protein by the gmLAB was confirmed by Western blotting. Bands corresponding to the signal peptide–conjugated form of mIL‐1Ra (24.5 kDa) and the signal peptide–cleaved form of mIL‐1Ra (21.8 kDa) were detected in the cell extract (Figure 1a). In the culture supernatant, only the signal peptide–cleaved form of mIL‐1Ra was detected (Figure 1b). By contrast, neither band was detected in the NZ‐VC or the NZ‐IL1Ra samples without nisin stimulation (Figure 1a,b).

### 3.2. Confirmation of IL‐1R Expression and rmIL‐1Ra Suppression of Inflammation‐Related Gene Expression in Pancreatic *β*‐Cells

To verify the effect of rmIL‐1Ra purified from NZ‐IL1Ra on gene expression in pancreatic cells, we first analyzed IL‐1R expression in a mouse pancreatic *β*‐cell line in the presence of IL‐1*β* (Figure 2a). In the IL‐1*β* − treatment group, IL‐1R expression on *β*‐TC‐tet cells was detected using an anti–IL‐1R Ab, but IL‐1R expression was not detected using this Ab in the no‐treatment group (Figure 2a: left). IL‐1R expression was also not detected using the control Ab (negative control) (Figure 2a). RT‐qPCR was performed to determine whether rmIL‐1Ra purified from NZ‐IL1Ra suppresses pancreatic inflammation (Figure 2b). IL‐1*β* induced inflammation in *β*‐TC‐tet cells, and RT‐qPCR was then performed using *Il6-*specific, *Tnfa-*specific, and *Nfkb*‐specific primers. The data are expressed as relative values versus the no‐treatment group (IL‐1*β* 0 ng/mL, IL‐1Ra 0 ng/mL). Compared with the positive control (IL‐1*β* 5 ng/mL, IL‐1Ra 0 ng/mL), expression of inflammation‐related genes (*Il6*, *Tnfa*, *Nfkb*) was significantly decreased in the presence of IL‐1Ra. Both commercially obtained IL‐1Ra and IL‐1Ra produced by the gmLAB had the same effect on mRNA expression.

Figure 2(a) Immunofluorescence staining of *β*‐TC‐tet cells to confirm IL‐1R expression. Cells were cultured for 2 h on cover‐glasses in a 24‐well plate at a density of 2 × 10^6^ cells per well and then stimulated for 24 h with or without IL‐1*β* (upper, no treatment; lower, IL‐1*β* treatment). IL‐1R expression was detected using a PE anti–mouse IL‐1R/CD121a antibody (IL‐1R Ab) and PE Armenian Hamster IgG isotype control antibody (Ctrl Ab, negative control). Nuclei were stained with DAPI. Blue and red staining show nuclei and IL‐1R, respectively. Scale bars = 50 *μ*m. (b) Results of RT‐qPCR analysis of the bioactivity of rmIL‐1Ra in terms of suppressing expression of inflammation‐related genes (*Il6*, *Tnfa*, *Nfkb*) in *β*‐TC‐tet cells. *β*‐TC‐tet cells were incubated with IL‐1*β* (0 or 5 ng/mL), commercially obtained mIL‐1Ra (commercial; 50 ng/mL), or purified IL‐1Ra (gmLAB; 50 or 100 ng/mL). Data are expressed as relative values versus no treatment (negative control; IL‐1*β*, 0 ng/mL; IL‐1Ra, 0 ng/mL) and as mean ± SD. Means denoted by different letters differed significantly (*p* < 0.05).

**Figure:**
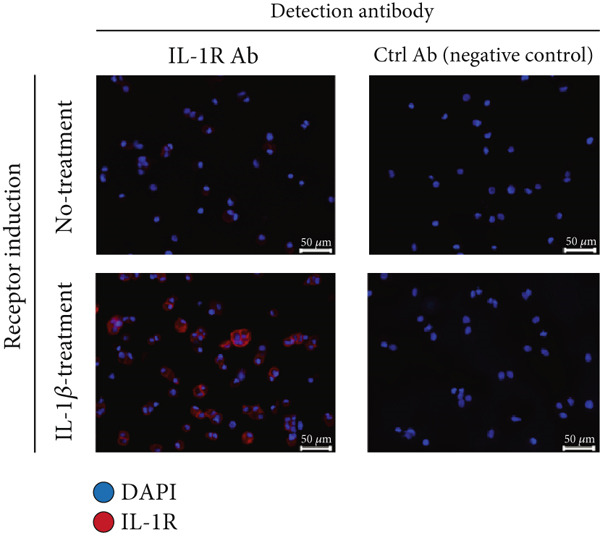
(a)

**Figure:**
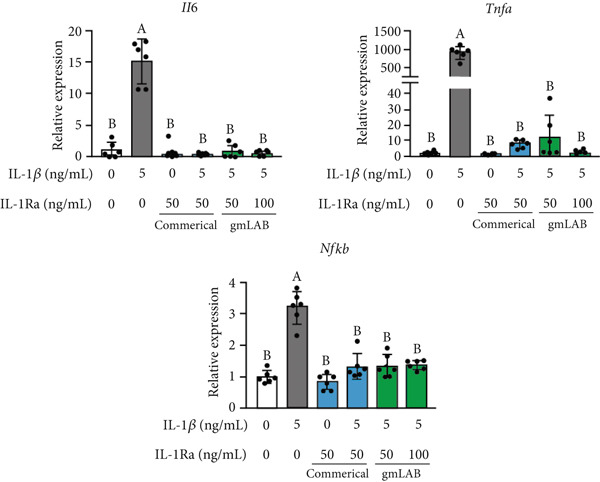
(b)

### 3.3. NZ‐IL1Ra Administration Tended to Improve Glucose Metabolism in Diet‐Induced Obese Mice

**Figure 3:**
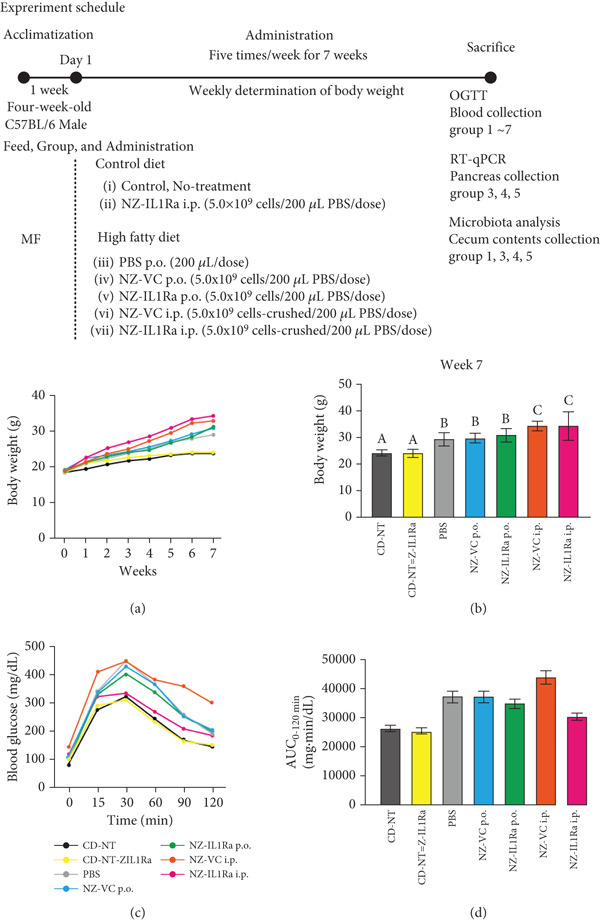
Experimental schedule, body weight, and OGTT results. Five‐week‐old mice were divided into seven groups: CD‐NT (control diet, no‐treatment), CD‐NZ‐IL1Ra (control diet, NZ‐IL1Ra p.o.), PBS (HFD, PBS p.o.), NZ‐VC p.o. (HFD, NZ‐VC p.o.), NZ‐IL1Ra p.o. (HFD, NZ‐IL1Ra p.o.), NZ‐VC i.p. (HFD, NZ‐VC i.p.), and NZ‐IL1Ra i.p. (HFD, NZ‐IL1Ra i.p.). Mice were administered 5.0 × 10^9^ cells/200 *μ*L of PBS, NZ‐IL1Ra, or NZ‐VC five times per week (total, 35 times) for 7 weeks via gavage or intraperitoneal injection. Body weight was measured weekly. OGTTs, collection of the pancreas for RT‐qPCR, and collection of cecum contents for microbiota analysis were performed 7 weeks after the first administration. (a) Change in body weight over the 7‐week administration period. Body weight was measured weekly. (b) Body weight at Week 7 (end of administration). (c) OGTT performed in C57BL/6 mice. After a 16‐h fast, blood glucose levels were measured in the fasting state (0 h) and at 15, 30, 60, 90, and 120 min after p.o. administration of glucose solution at 2 g/kg. CD‐NT: *n* = 12; CD‐NZ‐IL1Ra: *n* = 3; PBS: *n* = 16; NZ‐VC p.o.: *n* = 1; NZ‐IL1Ra p.o.: *n* = 14; NZ‐VC i.p.: *n* = 3; and NZ‐IL1Ra i.p.: *n* = 3. (d) Area under the OGTT curve from 0 to 120 min (AUC_0-120 min_) for each group. Data are expressed as mean ± SD. Means denoted by different letters differed significantly (*p* < 0.05).

To examine the effect of NZ‐IL1Ra on glucose metabolism, mice with diet‐induced obesity were treated with NZ‐IL1Ra and then subjected to an OGTT (Figure 3 shows the treatment schedule and experimental groups). Body weight was measured weekly. The OGTT was performed 7 weeks after the first administration of NZ‐IL1Ra. Comparing body weight at 7 weeks after first administration with that of mice in the CD groups (CD‐NT and CD‐NZ‐IL1Ra), the body weight of the PBS group was significantly increased (Figure 3a,b). In addition, OGTT (Figure 3c) and AUC_0-120 min_ (Figure 3d, Supporting information 1a) indicated that there was a significant increase in the blood glucose concentration in the PBS group compared with the CD‐NT group (Supporting information 1b). A significant decrease in the AUC was observed in the NZ‐IL1Ra p.o. and NZ‐IL1Ra i.p. groups compared with the PBS group (Supporting information 1c). The blood glucose concentration (particularly at 30 min, Supporting information 2a,b) in the NZ‐IL1Ra p.o. and NZ‐IL1Ra i.p. groups was significantly lower than that of the mice in the PBS group (Supporting information 2b). In addition, the extent of the decrease in blood glucose level (mean ± SD) was greater in the NZ‐IL1Ra i.p. group (332.3 ± 36.9 mg/dL) than in the NZ‐IL1Ra p.o. group (400.5 ± 41.5 mg/dL).

### 3.4. Difference in the Concentration of Pancreatic rmIL‐1Ra Between p.o. and i.p. Administration

To confirm the differences in rmIL‐1Ra kinetics observed with differing routes of administration, we measured the mIL‐1Ra concentration in the pancreas after NZ‐IL1Ra administration (Figure 4). A significant difference in mIL‐1Ra concentration in the pancreas (*p* < 0.0001) was also observed between p.o. and i.p. administration.

Figure 4(a) Schedule for comparing the pancreatic mIL‐1Ra concentration between p.o. and i.p. administration. NZ‐IL1Ra was administered five times at 30‐min intervals. Approximately 1.5 h after the fifth administration, the mice were sacrificed, and the pancreas was collected. (b) mIL‐1Ra concentration in the pancreas of the no treatment (gray), NZ‐IL1Ra p.o. (green), and NZ‐IL1Ra i.p. (red) groups. The concentration of mIL‐1Ra was measured, and the amount of mIL‐1Ra in the same weight of total protein was calculated.

**Figure:**
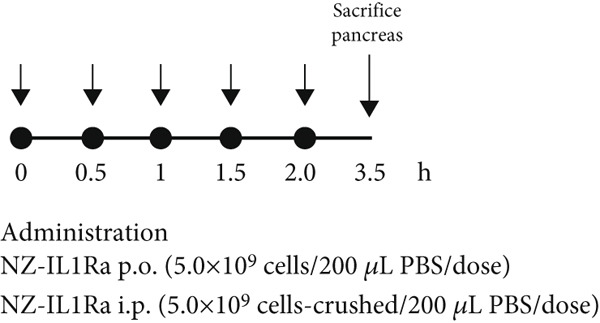
(a)

**Figure:**
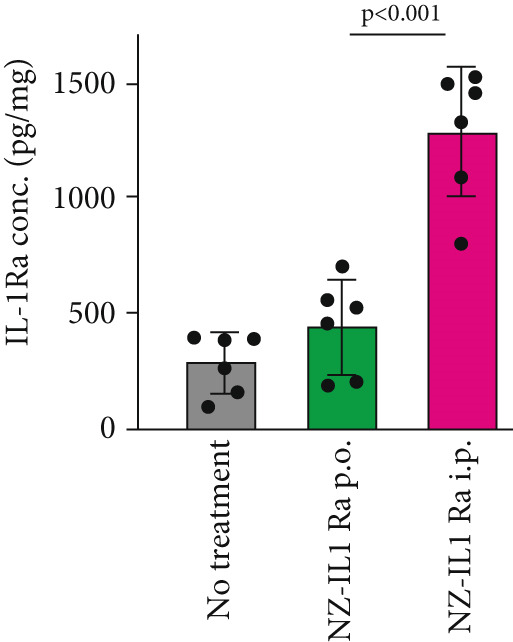
(b)

### 3.5. NZ‐IL1Ra Suppresses Expression of Inflammation‐Related Genes in the Pancreas

**Figure 5:**
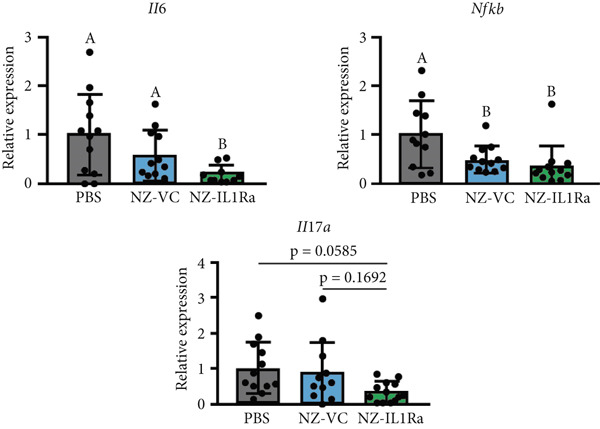
RT‐qPCR analysis of the PBS, NZ‐VC p.o., and NZ‐IL1Ra p.o. groups to evaluate the suppressive effect of NZ‐IL1Ra on the expression of inflammation‐related genes (*Il6*, *Nfkb*, *Il17a*) in the pancreas of C57BL/6 obesity model mice. Pancreas samples were collected at the end of the administration period. Data are expressed as relative values versus those of the PBS control group. Means denoted by different letters differed significantly (*p* < 0.05).

We also evaluated the effect of p.o. administration of NZ‐IL1Ra on gene expression in the pancreas. mRNA expression (pancreas: *Il6*, *Nfkb*, *Il17a*) was assessed using RT‐qPCR (Figure 5). Expression levels of *Il6* mRNA in the pancreas relative to levels in control mice that received PBS were significantly decreased in the NZ‐IL1Ra group compared with the NZ‐VC group, but there were no significant differences in expression levels of *Nfkb* between the NZ‐IL1Ra and NZ‐VC groups. Although no significant differences in *Il17a* expression levels were detected, we observed a notable decreasing trend in the NZ‐IL1Ra group compared with the NZ‐VC group.

### 3.6. Changes in the Intestinal Microbiome Following NZ‐IL1Ra Administration

We investigated whether NZ‐IL1Ra administration induced changes in the constituents of the intestinal microbiome using 16S V3‐V4 rRNA gene sequencing. In terms of *α*‐diversity, no significant difference was detected between the NZ‐IL1Ra and NZ‐VC groups in Faith′s PD (Figure 6a). Differences in diversity between samples were also assessed using *β*‐diversity analysis to compare the bacterial compositions of the CD‐NT, PBS, NZ‐VC, and NZ‐IL1Ra groups (Figure 6b). The principal coordinate plot revealed a separation in the gut microbiota composition (Figure 6b left), and significantly different distances in gut microbiota composition were observed between the NZ‐IL1Ra and the control (*p* < 0.05), PBS (*p* < 0.05), and NZ‐VC (*p* < 0.05) groups (Figure 6b, Supporting information 3). Significant differences in the abundance of various taxa were observed between the PBS, NZ‐VC, and NZ‐IL1Ra groups (Figure 6c). In the NZ‐IL1Ra group, Lachnospiraceae *A2* and Lachnospiraceae *Acetatifactor* were more abundant compared with the PBS and NZ‐VC groups (Figure 6c).

Figure 6Oral administration of NZ‐IL1Ra altered the gut microbiota. Mice were divided into four groups, CD‐NT (control diet, no treatment, *n* = 6), PBS (PBS p.o., *n* = 7), NZ‐VC (NZ‐VC p.o., *n* = 6), and NZ‐IL1Ra (NZ‐IL1Ra p.o., *n* = 6). Cecal content samples were collected at the end of the administration period. (a) Boxplots depicting *α*‐diversity (Faith′s PD) indices of the microbiota composition in mice. (b) *β*‐diversity of the intestinal microbiota composition in mice, displayed using Bray–Curtis distances. Circles around the dots shows 95% confidential intervals. Differences in distance of microbiota composition between the control and PBS groups and NZ‐VC and NZ‐IL1Ra groups. Means denoted by different letters differed significantly (*p* < 0.05). (c) Taxa in the NZ‐IL1Ra group that exhibited a significant increase (*p* < 0.05) compared with the CD‐NT, PBS, and NZ‐VC groups. Means denoted by different letters differed significantly (*p* < 0.05).

**Figure:**
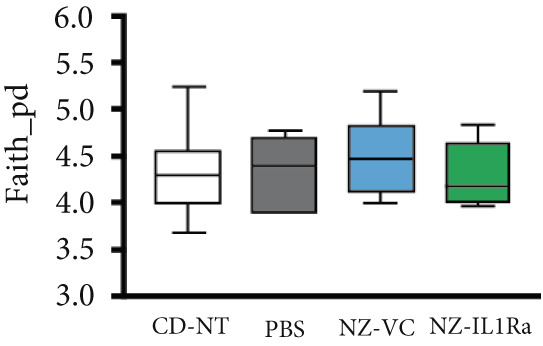
(a)

**Figure:**
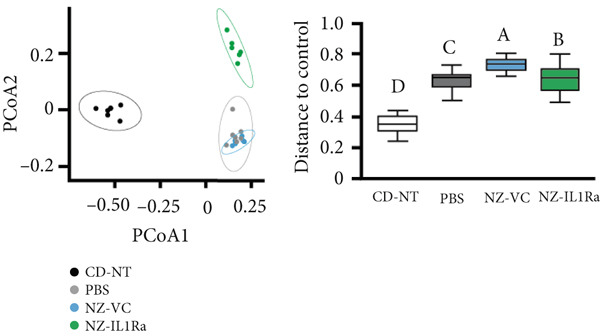
(b)

**Figure:**
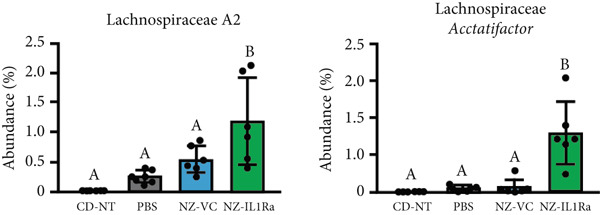
(c)

## 4. Discussion

IL‐1*β* is a pivotal pro‐inflammatory mediator of all inflammatory processes in a variety of tissues, and it is typically produced by adipocytes, macrophages, and *β*‐cells of pancreatic islets. High glucose concentrations and prolonged exposure to glucose induce the release of IL‐1*β* from pancreatic islets [24]. The cytotoxic effects of IL‐1*β* on *β*‐cells have been reported [4, 25]. This role in mediating cytotoxicity of IL‐1*β* was also confirmed by the expression of IL‐1*β* under hyperglycemic conditions by pancreatic *β*‐cells in different animal models [26–28]. These studies suggested that IL‐1*β* signaling must be controlled, and blocking the binding of IL‐1*β* to its receptor, IL‐R Type I, has been proposed as the most suitable approach. For example, a clinical trial showed that blockade of IL‐1*β* using anakinra, an IL‐1Ra, in the treatment of inflammatory diseases improves glycemia and *β*‐cell secretory function and reduces markers of systemic inflammation [14]. These reports indicate that inhibition of IL‐1*β* signaling using an IL‐1Ra may improve *β*‐cell function, prevent pancreatic islet cell apoptosis, and ameliorate the effects of hyperglycemia. Thus, we focused on NZ‐IL1Ra, which was previously constructed as an IL‐1Ra–hypersecreting gmLAB, and examined the effects of NZ‐IL1Ra on glucose metabolism using a mouse pancreatic *β*‐cell line in vitro and on diet‐induced T2DM C57BL/6 mice in vivo.

In this study, we initially confirmed recombinant protein expression by NZ‐IL1Ra using Western blotting. Next, we investigated whether rmIL‐1Ra produced by NZ‐IL1Ra suppresses inflammation in mouse pancreas. We first confirmed IL‐1R expression by *β*‐TC‐tet mouse pancreatic *β*‐cells using immunofluorescence staining with and without IL‐1*β* signaling. Expression of the receptor was detected only under the condition of IL‐1*β* induction and IL‐1R Ab detection, demonstrating that IL‐1*β* signaling induces IL‐1R expression in mouse pancreatic *β*‐cells. The cytotoxic effects of IL‐1*β* on *β*‐cells of pancreatic islets have also been extensively studied [25]; therefore, we next examined whether rmIL‐1Ra suppresses the mRNA expression of inflammation‐related genes. The relative expression levels of *Il6*, *Tnfa*, and *Nfkb* were significantly decreased in cells incubated with IL‐1*β* or purified IL‐1Ra compared with cells incubated with only IL‐1*β*. Similarly, a decrease in the mRNA expression of inflammation‐related genes was observed using commercially obtained mIL‐1Ra, indicating that mIL‐1Ra purified from gmLAB and commercially available mIL‐1Ra exert identical effects on pancreatic islet *β*‐cells.

Although numerous factors affect the progression of T2DM, pro‐inflammatory cytokines such as IL‐6 and TNF‐*α* play particularly important roles. For example, a study has shown that mice lacking TNF‐*α* or TNF receptors exhibit improved insulin sensitivity in both dietary and genetic models of obesity [29]. IL‐6 plays roles not only in inflammatory signaling pathways but also in insulin resistance in hepatocytes [30] and adipocytes [31]. Furthermore, IL‐1*β* derived from islet macrophages impairs insulin secretion, thereby contributing to the development of glucose intolerance and T2DM. Thus, the observed decrease in *Nfkb* mRNA expression suggests that mIL‐1Ra blocks *Nfkb* signaling [32] to prevent tissue inflammation caused by IL‐1*β*. In vitro experiment data suggest that mIL‐1Ra derived from NZ‐IL1Ra improves risk factors associated with the development of T2DM.

Although our data from analyses using a mouse pancreatic *β*‐cell line suggest that NZ‐IL1Ra improves risk factors associated with the development of T2DM, we do not know whether NZ‐IL1Ra affects the pathogenesis of obesity‐related insulin resistance and T2DM in vivo. We therefore administered NZ‐IL1Ra to mice for 7 weeks to clarify whether NZ‐IL1Ra improves glucose metabolism. A syndrome resembling T2DM has been reported in studies using several animal models, such as *Psammomys obesus* [26], Goto–Kakizaki rats [27], and human islet amyloid polypeptide transgenic rats [28]. Comparing glucose metabolism in C57BL/6 mice with that in other animal models is a particularly useful approach for studying human metabolic syndrome because these mice develop a syndrome involving obesity, hyperinsulinemia, hyperglycemia, and hypertension when allowed ad libitum access to a HFD [33, 34]. Thus, we examined the effect of NZ‐IL1Ra administration using diet‐induced T2DM C57BL/6 mice.

After 7 weeks, mice with diet‐induced obesity showed significant increases in body weight (Figure 3a,b, CD‐NT vs. PBS) and AUC_0-120 min_ (Figure 3c,d, Supporting information 1b), as expected. OGTT results (Figure 3c,d) showed that p.o. and i.p. administration of NZ‐IL1Ra induced a significant suppression of the blood glucose level when compared with the PBS group at 30 min after glucose administration (Supporting information 2). However, in our OGTTs, the AUC_0-120 min_ (Supporting information 1a) and blood glucose level (Supporting information 2) indicated that i.p. was more successful than p.o. in terms of the degree of blood glucose lowering. We speculate that the difference could be attributed to differences in pharmacokinetics with each of the administration methods. We thus confirmed the difference in mIL‐1Ra concentration in the pancreas with i.p. versus p.o. administration (Figure 4). These findings indicate that mIL‐1Ra is transported to the pancreas more efficiently via i.p. delivery than via oral gavage. Substances administered via the i.p. route are typically absorbed directly through the subperitoneal capillaries. This could explain the difference in blood glucose levels between the p.o. and i.p. administration groups [35]. Thus, NZ‐IL1Ra decreases blood glucose levels, but the extent depends on the route of administration. Next, we investigated the effect of oral administration on the expression of inflammation‐related genes in the pancreas (Figure 3 experimental schedule, Groups 3, 4, and 5) (Figure 5). The expression levels of *Il6* and *Nfkb* indicated that p.o. administration of NZ‐IL1Ra inhibited pancreatic inflammation. We did not observe a significant decrease in *Il17a* expression, but a trend toward lower gene expression was observed in the NZ‐IL1Ra p.o. group compared with the PBS and NZ‐VC p.o. groups. IL‐17A plays a critical role in host defense against a variety of microbial pathogens as well as in mediating tissue inflammation. In host defense, IL‐17A exerts predominantly beneficial effects against infections caused by extracellular bacteria and fungi; however, this cytokine also plays critical roles in multiple autoimmune diseases [36]. These reports indicate that IL‐17 family cytokines appear to play a pathologic role in diabetic model mice, and IL‐17A deficiency has been shown to ameliorate diabetes [37]. Thus, oral administration of NZ‐IL1Ra exerts a slight suppressive effect on pancreatic inflammation and blood glucose levels in mice.

Recent advances in detailed high‐throughput sequencing for use in areas such as metatranscriptomics, metagenomics, proteomics, and metabolomics, combined with advances in bioinformatics, have shed more light on the impact of the gut microbiome composition and its effect on systemic metabolic processes, leading to an increase in research into the role of the gut microbiome in T2DM [38, 39]. We therefore investigated whether NZ‐IL1Ra administration results in alterations in the constituents of the intestinal microbiome using 16S rRNA gene sequence. These analyses revealed that although *α*‐diversity was not affected, *β*‐diversity was significantly affected in the NZ‐IL1Ra group compared with the control, PBS, and NZ‐VC groups. Importantly, the relative outgrowth of Lachnospiraceae *A2* and Lachnospiraceae *Acetatifactor* in mice was specifically associated with NZ‐IL1Ra administration.

*Acetatifactor* was recently identified as a lithocholic acid–producing bacterium that expresses the enzyme 7*α*‐dehydroxylase and converts primary bile acids, cholic acid, and chenodeoxycholic acid into deoxycholic acid [40]. Bile acids play roles in the regulation of lipid, glucose, and energy homeostasis through activation of farnesoid X receptor and G protein–coupled bile acid receptor‐1 [41, 42]. Thus, *Acetatifactor* might play a critical regulatory role in improving T2DM. Although no significant difference in Lachnospiraceae *A2* abundance was observed between the CD‐NT and PBS groups in this study, the abundance in the NZ‐IL1Ra group was significantly increased compared with the other groups. A previous report showed that the abundance of Lachnospiraceae *A2* decreases in mice fed a HFD [43], suggesting that an increase in the Lachnospiraceae *A2* abundance may be associated with metabolic homeostasis. Although our findings contradict this possibility, as no significant difference in Lachnospiraceae *A2* abundance was observed between the HFD and CD groups, the elevated abundance in the NZ‐IL1Ra group remains intriguing and may still indicate that these organisms play a role in maintaining metabolic homeostasis. The results of our microbiota analyses thus indicate that oral administration of NZ‐IL1Ra changes the composition of the microbiome in diet‐induced T2DM mice and that the pathway to target for improving glucose metabolism is separate from the pathway to target for suppressing organ inflammation.

Although an effect of p.o. administration of NZ‐IL1Ra on glucose metabolism was demonstrated in the present study, the extent was lower than that of i.p. NZ‐IL1Ra administration. Therefore, further investigations of NZ‐IL1Ra administration via the p.o. route are warranted to facilitate more effective oral delivery (e.g., twice daily p.o. and for a longer term than 7 weeks).

## 5. Limitations

In this study, we examined the effect of NZ‐IL1Ra administration using only male mice. Although a previous study showed that male C57BL/6 mice gain significantly more weight on a HFD than female mice [44], suggesting that male C57BL/6 mice are a better model, how the sex difference affects glucose metabolism was not elucidated. The lack of confirmation of a sex‐related difference is a limitation of this work. Another limitation includes the small size of the microbiota analysis group, which reduces the confidence in our conclusions. Thus, the connection between microbial shifts and improved metabolism is speculative rather than definitive.

## 6. Conclusions

In this study, we used *β*‐TC‐tet cell lines to confirm that NZ‐IL1Ra secretes rmIL‐1Ra and that purified rmIL‐1Ra exhibits anti‐inflammatory activity in the pancreas. Subsequent in vivo OGTT results showed improvements in blood glucose levels in mice administered NZ‐IL1Ra via the i.p. route; however, the levels were slightly improved in mice administered NZ‐IL1Ra via the p.o. route. Our analysis indicated that the difference might be attributable to the kinetics of rmIL‐1Ra. Moreover, RT‐qPCR analyses demonstrated that p.o. administration of NZ‐IL1Ra exerts a slight suppressive effect on pancreatic inflammation in mice. Finally, we showed that p.o. administration of NZ‐IL1Ra leads to an increase in the abundance of Lachnospiraceae *A2* and *Acetatifactor* species, which play roles in improving glucose metabolism in the mouse intestine. Taken together, our results suggest that administration of NZ‐IL1Ra may be useful for improving glucose metabolism.

## Ethics Statement

All animal procedures were performed according to the regulations for Animal Experimentation of Shinshu University (Approval No. 023095).

## Disclosure

All authors provided feedback on previous manuscript versions and have read and approved the final version.

## Conflicts of Interest

T.F. and T.T. are members of BIOSIS Lab. Co., Ltd. The remaining authors declare that the research was conducted without commercial or financial relationships that could be construed as a potential conflict of interest.

## Author Contributions

N.N., M.Y., S.Y., M.K., A.M., and F.N. prepared the materials, conducted the experiments, and collected and analyzed the data. T.Sa. and T.Sh. designed the research and contributed to data interpretation. T.F. and T.T. were responsible for gut microbiota analysis. M.Y. drafted the initial manuscript, with contributions from A.M. and F.N., and T.Sh. provided a comprehensive review. M.Y. and N.N. contributed equally to this work.

## Funding

This work was supported by the Education and Research Support Fund from Shinshu University, 2019–2024; J‐PEAKS, JPJS00420230007.

## Supporting information


**Supporting Information** Additional supporting information can be found online in the Supporting Information section. Supporting information 1. Area under the blood glucose concentration curve from 0 min to 120 min and results of statistical analyses (a) calculated AUC, (b) analysis CD‐NT vs. PBS, and (c) multiple comparisons. The AUC_0-120 min_ was calculated using the GraphPad Prism software (GraphPad). the Welch′s *t* test was conducted between CD‐NT group and PBS group. There was significant increase of the blood glucose concentration in PBS group compared with the CD‐NT group. Dunnett′s multiple comparisons test was conducted among HFD group (PBS, NZ‐VC p.o., NZ‐IL1Ra p.o., NZ‐VC i.p., and NZ‐IL1Ra i.p.) using the PBS group as a control. Compared to PBS group, the AUC of NZ‐IL1Ra p.o. and NZ‐IL1Ra i.p. significantly decreased. On the other hand, there was significant increase in that of NZ‐VC i.p. ∗∗*p* < 0.005, ∗∗∗∗*p* < 0.0001. Supporting information 2. (a) The blood glucose concentration at 30 min. (b) Two‐way repeated‐measures mixed‐effect models with group × time of the blood glucose concentration of OGTT. The blood glucose concentration in OGTT at 30 min was shown. Two‐way repeated‐measures mixed‐effect models with group × time and Dunnett′s multiple comparisons test was conducted among HFD group (PBS, NZ‐VC p.o., NZ‐IL1Ra p.o., NZ‐VC i.p., and NZ‐IL1Ra i.p.) using the PBS group as a control. In 0 min, there was significant increase of blood glucose concentration in NZ‐VC i.p. group compared with PBS group, which suggested there was a tendency that animals have high blood glucose concentration in NZ‐VC i.p. group. At 30 min, the blood glucose concentration of NZ‐IL1Ra p.o. and NZ‐IL1Ra i.p. significantly decreased compared with PBS group. ∗*p* < 0.05. Supporting information 3. Differential taxa in *β*‐diversity were analyzed using adonis (PERMANOVA) in Qiime2. Sample size, the number of times of permutation test (Permutations), pseudo‐*F* value, *p* value, and *q* value are shown.

## Data Availability

The datasets used and analyzed during the current study are available from the corresponding author on reasonable request. The composition of the intestinal microbiota determined using 16S V3‐V4 rRNA gene sequencing, which supports the findings of this study, was deposited in the DDBJ under bio‐project accession number PRJDB20232.
