# Supplementary material for: Genetically Modified Lactococcus lactis Hypersecreting IL‐1Ra Improves Glucose Metabolism and Modulates the Gut Microbiota in an Obese Mouse Model
Source: J Diabetes Res. 2026 Feb 1;2026:6006491. doi: 10.1155/jdr/6006491 (PMC12862108; doi:10.1155/jdr/6006491)
Supplement: Supplementary file 1 — Supporting Information Additional supporting information can be found online in the Supporting Information section. Supporting information 1. Area under the blood glucose concentration curve from 0 min to 120 min and results of statistical analyses (a) calculated AUC, (b) analysis CD‐NT vs. PBS, and (c) multiple comparisons. The AUC0-120 min was calculated using the GraphPad Prism software (GraphPad). the Welch′s t test was conducted between CD‐NT group and PBS group. There was significant increase of the blood glucose concentration in PBS group compared with the CD‐NT group. Dunnett′s multiple comparisons test was conducted among HFD group (PBS, NZ‐VC p.o., NZ‐IL1Ra p.o., NZ‐VC i.p., and NZ‐IL1Ra i.p.) using the PBS group as a control. Compared to PBS group, the AUC of NZ‐IL1Ra p.o. and NZ‐IL1Ra i.p. significantly decreased. On the other hand, there was significant increase in that of NZ‐VC i.p. ∗∗p < 0.005, ∗∗∗∗p < 0.0001. Supporting information 2. (a) The blood glucose concentration at 30 min. (b) Two‐way repeated‐measures mixed‐effect models with group × time of the blood glucose concentration of OGTT. The blood glucose concentration in OGTT at 30 min was shown. Two‐way repeated‐measures mixed‐effect models with group × time and Dunnett′s multiple comparisons test was conducted among HFD group (PBS, NZ‐VC p.o., NZ‐IL1Ra p.o., NZ‐VC i.p., and NZ‐IL1Ra i.p.) using the PBS group as a control. In 0 min, there was significant increase of blood glucose concentration in NZ‐VC i.p. group compared with PBS group, which suggested there was a tendency that animals have high blood glucose concentration in NZ‐VC i.p. group. At 30 min, the blood glucose concentration of NZ‐IL1Ra p.o. and NZ‐IL1Ra i.p. significantly decreased compared with PBS group. ∗p < 0.05. Supporting information 3. Differential taxa in β‐diversity were analyzed using adonis (PERMANOVA) in Qiime2. Sample size, the number of times of permutation test (Permutations), pseudo‐F value, p value, and q value are [file JDR-2026-6006491-s001.zip › Supplementary materials_JDR_revision2.docx]

**Supplementary materials**

**Supplementary material 1**

**Area under the blood glucose concentration curve from 0 min to 120 min and results of statistical analyses**

1. Calculated AUC

|  | **CD-NT** | **CD-NZ-IL1Ra** | **PBS** | **NZ-VC p.o.** | **NZ-IL1Ra p.o.** | **NZ-VC i.p.** | **NZ-IL1Ra i.p.** |
| --- | --- | --- | --- | --- | --- | --- | --- |
| **Total Area**  **(mg・min/dL)** | 26521 | 25890 | 37511 | 37336 | 35150 | 44150 | 30105 |
| **Std. Error** | 1273 | 738.9 | 1800 | 2113 | 1607 | 2441 | 1258 |
| **95% Confidence Interval** | 24026  to  29015 | 24442  to  27338 | 33983  to  41040 | 33195  to  41476 | 32001  to  38300 | 39367  to  48933 | 27639  to  32571 |

1. Analysis CD-NT vs PBS

| **Unpaired t test with Welch's correction** |  |
| --- | --- |
| P value | <0.0001 |
| P value summary | **** |
| Significantly different (P < 0.05)? | Yes |

1. Multiple comparisons

| **Dunnett's multiple comparisons test** | Summary | P Value |
| --- | --- | --- |
| **PBS vs. NZ-VC p.o.** | ns | 0.9974 |
| **PBS vs. NZ-IL1Ra p.o.** | ** | 0.0036 |
| **PBS vs. NZ-VC i.p.** | **** | <0.0001 |
| **PBS vs. NZ-IL1Ra i.p.** | **** | <0.0001 |

**a** The AUC_0-120 min_ was calculated using the GraphPad Prism software (GraphPad). **b** the Welch’s t test was conducted between CD-NT group and PBS group. There was significant increase of the blood glucose concentration in PBS group compared with the CD-NT group. **c** Dunnett's multiple comparisons test was conducted among HFD group (PBS, NZ-VC p.o., NZ-IL1Ra p.o., NZ-VC i.p., and NZ-IL1Ra i.p.) using the PBS group as a control. Compared to PBS group, the AUC of NZ-IL1Ra p.o. and NZ-IL1Ra i.p. significantly decreased. On the other hand, there was significant increase in that of NZ-VC i.p..

**Supplementary material 2**

a The blood glucose concentration at 30 minutes

b Two-way repeated-measures mixed-effect models with group x time of the blood glucose concentration of OGTT

| **Dunnett's multiple comparisons test** | Summary | P Value |
| --- | --- | --- |
| **0 minute** | | |
| **PBS vs. NZ-VC p.o.** | ns | 0.9955 |
| **PBS vs. NZ-IL1Ra p.o.** | ns | 0.7590 |
| **PBS vs. NZ-VC i.p.** | * | 0.0262 |
| **PBS vs. NZ-IL1Ra i.p.** | ns | 0.8158 |
| **15 minutes** | | |
| **PBS vs. NZ-VC p.o.** | ns | >0.9999 |
| **PBS vs. NZ-IL1Ra p.o.** | ns | 0.7930 |
| **PBS vs. NZ-VC i.p.** | ns | 0.4166 |
| **PBS vs. NZ-IL1Ra i.p.** | ns | 0.8013 |
| **30 minutes** | | |
| **PBS vs. NZ-VC p.o.** | ns | 0.7283 |
| **PBS vs. NZ-IL1Ra p.o.** | * | 0.0157 |
| **PBS vs. NZ-VC i.p.** | ns | >0.9999 |
| **PBS vs. NZ-IL1Ra i.p.** | * | 0.0454 |
| **60 minutes** | | |
| **PBS vs. NZ-VC p.o.** | ns | >0.9999 |
| **PBS vs. NZ-IL1Ra p.o.** | ns | 0.4259 |
| **PBS vs. NZ-VC i.p.** | ns | 0.9951 |
| **PBS vs. NZ-IL1Ra i.p.** | ns | 0.0711 |
| **90 minutes** | | |
| **PBS vs. NZ-VC p.o.** | ns | 0.9993 |
| **PBS vs. NZ-IL1Ra p.o.** | ns | 0.9908 |
| **PBS vs. NZ-VC i.p.** | ns | 0.2007 |
| **PBS vs. NZ-IL1Ra i.p.** | ns | 0.1154 |
| **120 minutes** | | |
| **PBS vs. NZ-VC p.o.** | ns | 0.9372 |
| **PBS vs. NZ-IL1Ra p.o.** | ns | 0.9996 |
| **PBS vs. NZ-VC i.p.** | ns | 0.2296 |
| **PBS vs. NZ-IL1Ra i.p.** | ns | 0.7104 |

**a** The blood glucose concentration in OGTT at 30 min was shown. **b** Two-way repeated-measures mixed-effect models with group x time and dunnett's multiple comparisons test was conducted among HFD group (PBS, NZ-VC p.o., NZ-IL1Ra p.o., NZ-VC i.p., and NZ-IL1Ra i.p.) using the PBS group as a control. In 0 min, there was significant increase of blood glucose concentration in NZ-VC i.p. group compared to PBS group which suggested there was a tendency that animals have high blood glucose concentration in NZ-VC i.p. group. At 30 min, the blood glucose concentration of NZ-IL1Ra p.o. and NZ-IL1Ra i.p. significantly decreased compared to PBS group.

**Supplementary material 3**

| **Group 1** | **Group 2** | **Sample size** | **Permutations** | **pseudo-F** | **p-value** | **q-value** |
| --- | --- | --- | --- | --- | --- | --- |
| CD-NT | PBS | 13 | 999 | 45.8594375528414 | 0.001 | 0.004 |
| CD-NT | NZ-VC p.o. | 12 | 999 | 48.2885020692299 | 0.006 | 0.006 |
| CD-NT | NZ-IL1Ra p.o | 12 | 999 | 46.4771983972172 | 0.005 | 0.006 |
| PBS | NZ-VC p.o. | 13 | 999 | 4.56921791268222 | 0.002 | 0.004 |
| PBS | NZ-IL1Ra p.o | 13 | 999 | 11.8622480977062 | 0.003 | 0.0045 |
| NZ-VC p.o. | NZ-IL1Ra p.o | 12 | 999 | 13.571225690887 | 0.002 | 0.004 |

Differential taxa in β-diversity were analyzed using adonis (PERMANOVA) in Qiime2. Sample size, the number of times of permutation test (Permutations), pseudo-F value, p-value, and q-value are shown.
